# AI-Assisted Design of 3D-Printed Lingual Indirect Bonding Trays: A Comparative Evaluation of Bracket Transfer Accuracy

**DOI:** 10.3390/jcm14124303

**Published:** 2025-06-17

**Authors:** Viet Hoang, Thi Quynh Trang Vuong, Phuong Huyen Nguyen, Nhu Hai Pham, Kim Loan Hoang, Thi Thu Trang Hoang, Tuan Khang Nguyen, Thi Hong Thuy Pham, Viet Anh Nguyen

**Affiliations:** 1 Department of Orthodontics and Pedodontics, Faculty of Dentistry, Van Lang University, Ho Chi Minh City 70000, Vietnam; viet.h@vlu.edu.vn; 2 Private Practice, Viet Anh Orthodontic Clinic, Hanoi 12015, Vietnam; quynhtrang@chinhnhavietanh.com; 3 Department of Pediatric Dentistry, National Hospital of Odonto-Stomatology, Hanoi 11017, Vietnam; huyenrangtreem@gmail.com; 4 Faculty of Dentistry, VNU Hanoi-University of Medicine and Pharmacy, Hanoi 11310, Vietnam; phamnhuhai@vnu.edu.vn; 5 School of Dentistry, Hanoi Medical University, Hanoi 11521, Vietnam; kimloan@hmu.edu.vn (K.L.H.); hoangthutrang@hmu.edu.vn (T.T.T.H.); 6 Faculty of Dental Medicine and Oral Health Sciences, McGill University, Montreal, QC H3A 0G4, Canada; tuan.k.nguyen@mail.mcgill.ca; 7 Department of Orthodontics, Hai Phong University of Medicine and Pharmacy, Hai Phong 04212, Vietnam; pthongthuy@hpmu.edu.vn; 8 Faculty of Dentistry, PHENIKAA University, Hanoi 12116, Vietnam

**Keywords:** indirect bonding, 3D printing, lingual brackets, artificial intelligence, digital workflow

## Abstract

**Objectives:** This study investigated the use of artificial intelligence (AI) in the design of lingual bracket indirect bonding trays and its association with bracket transfer accuracy using three-dimensional (3D) printing. **Methods:** Digital impressions of patient’s dental arches were captured using an intraoral scanner, and orthodontic setups were virtually constructed. Brackets were virtually positioned in their ideal locations using the digital setups guided by virtual archwire templates. Indirect bonding trays were automatically generated using the AI-powered Auto Creation function of the Medit Splints application, which analyzes anatomical features to streamline design. Bracket transfer accuracy was evaluated in vivo by comparing planned and actual bracket positions across grouped and individual tray configurations. Linear and angular deviations were measured using conventional 3D inspection software. **Results:** Most bracket transfer errors were within clinically acceptable thresholds, although torque accuracy remained suboptimal. Grouped trays generally exhibited greater precision than individual trays in several dimensions. **Conclusions:** These findings support the application of AI-assisted design tools to enhance digital workflows and improve consistency in appliance fabrication.

## 1. Introduction

Indirect bonding trays have been extensively studied and have demonstrated the ability to improve the accuracy of bracket placement, particularly in lingual orthodontics. The challenging intraoral environment of lingual orthodontics, with its limited visibility and access, makes indirect bonding especially advantageous [1]. Advances in digital technology and three-dimensional (3D) printing now allow for the fabrication of indirect bonding trays on 3D-printed models or through direct printing. Notably, directly 3D-printed trays have exhibited superior bracket transfer accuracy compared to vacuum-formed trays [2]. This enhanced accuracy likely stems from eliminating potential errors introduced during the intermediate steps of model 3D printing and vacuum forming [3,4].

Traditional analog methods for the indirect bonding of lingual brackets involve numerous laboratory steps. These typically include sectioning study models into individual teeth for orthodontic setup. Because lingual brackets are positioned on these separated teeth, their precise placement cannot be directly transferred back to the original model. Consequently, indirect bonding trays are fabricated for each individual tooth, and these trays are then used for transferring one tooth at a time to the patient’s mouth [5]. This single-tooth transfer method may reduce accuracy due to the limited anatomical landmarks available for reference, particularly for smaller teeth [6]. In contrast, digital orthodontic setup and bracket placement allow brackets to be virtually positioned on the teeth in their aligned positions. Subsequently, these entire setups, with brackets fixed to the teeth, are digitally moved back to the original malocclusion state, enabling the fabrication of grouped indirect bonding trays.

More recently, artificial intelligence (AI) has begun transforming digital dentistry by enhancing automation and streamlining design processes. In orthodontics, AI-powered applications are emerging in areas such as tooth segmentation and appliance design [7]. One such application is Medit Splints, which features an AI-powered Auto Creation tool that analyzes the morphology of anterior and posterior teeth to automatically generate splints or tray geometries.

In terms of tray configuration, grouped trays are hypothesized to improve transfer accuracy by preventing mesiodistal displacement [6]. However, the reduced inter-bracket distance inherent to lingual orthodontics may hinder the full seating of grouped trays, particularly in cases with crowding or when using rigid tray materials due to potential undercuts that obstruct complete tray adaptation. Several recent investigations have explored other aspects of tray design and fabrication to improve indirect bonding outcomes. For instance, Karabiber and Eglenen compared two types of 3D-printed tray designs—a full-coverage “shell” versus a minimalist “bar” design—and found no significant difference in overall bracket transfer accuracy between these design approaches [8]. Schwärzler et al. examined the effect of tray material rigidity by comparing hard versus soft resin trays in a randomized clinical trial; they reported that both materials yielded similarly high accuracy, with all mean transfer errors within clinically acceptable limits [9]. Additionally, an in vivo lingual bonding study by Anh et al. using double vacuum-formed trays noted that torque was the least accurately transferred dimension, underscoring the difficulty of achieving perfect angular accuracy with current methods [6]. However, none of these studies addressed the question of whether using a multi-tooth tray (grouped tray) versus individual single-tooth trays results in different accuracy outcomes. This gap in the literature is especially pertinent to lingual orthodontics, where limited space and complex tooth anatomy could influence tray fit and bracket positioning. To date, no direct comparison of the transfer accuracy between grouped and individual tray configurations has been published.

Therefore, this study aims to evaluate the in vivo transfer accuracy of lingual brackets using 3D-printed trays designed with AI-assisted tools. Additionally, the clinical accuracy of grouped versus individual tray configurations was compared to assess whether tray configuration has an impact on bracket placement precision. The first null hypothesis was that bracket transfer errors using AI-assisted design trays would not fall within clinically acceptable thresholds. The second null hypothesis was that there would be no significant difference in bracket transfer accuracy between the two tray types.

## 2. Materials and Methods

### 2.1. Subjects

This study adhered to the STROBE guidelines. The study protocol received ethical approval from the Hanoi Medical University Institutional Review Board (HMUIRB970). All study methods were conducted in accordance with the relevant guidelines and regulations. Twenty-two consecutive patients undergoing lingual orthodontic treatment in both arches were recruited from a private practice (Nam Tu Liem, Hanoi) between August 2023 and December 2023. The inclusion criteria included permanent dentition with normal crown anatomy and mild to moderate crowding.

Sample size calculation was based on the mean torque transfer errors of two 3D-printed indirect bonding trays, 0.83° ± 0.46° and 1.12° ± 0.50°, reported by Wang et al. [10]. Torque values were selected for this calculation because previous studies on the transfer accuracy of indirect bonding trays generally indicated that torque was the least accurately transferred dimension [2,3,6,10]. The calculation determined that a minimum of 44 brackets would need to be analyzed to achieve a significance level of 0.05 and a power of 80% to detect significant differences.

### 2.2. Indirect Bonding Tray Design

**Figure 1:**
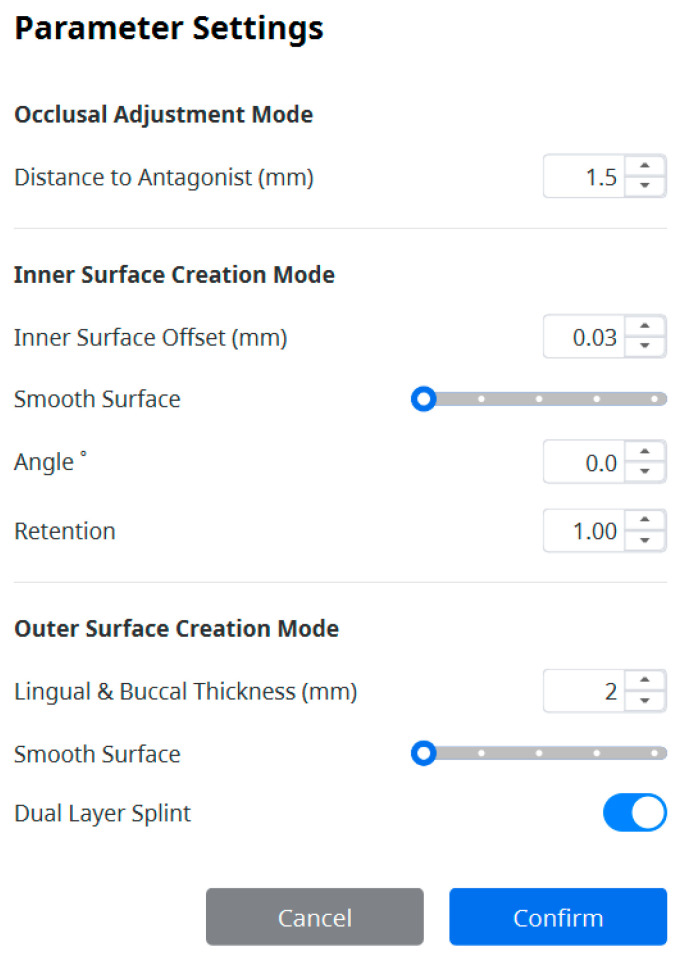
Parameter settings for AI-assisted design of indirect bonding trays using the Medit Splints application version 1.1.

Digital impressions of the patients’ dentitions were taken with an intraoral scanner (i700, Medit, Seoul, Republic of Korea). Following the workflows presented by Nguyen et al. [1], tooth segmentation, virtual orthodontic setup creation, bracket placement, and working model exportation were performed. Brackets were initially positioned in the planned final alignment using a virtual archwire template, ensuring that the horizontal slots of all brackets were positioned within the same reference plane. The ideal bracket positions were then reverted to the pretreatment tooth alignment to generate the working models. These models were imported into an AI-assisted automatic splint design application (Medit Splints, version 1.1, Medit, Republic of Korea) to fabricate the indirect bonding trays. The splints were created using the following parameters: dual-layer mode enabled, inner surface offset set to 0.03 mm, angle 0°, retention level 1 (maximum), and total lingual and buccal thickness of 2.0 mm, yielding an exact inner tray thickness of approximately 1.0 mm (Figure 1). The smooth surface option was disabled to avoid potential loss of bracket retention and to minimize uneven tray thickness. Dual-layer mode was selected instead of single-layer mode because the latter automatically smooths the outer surface, which may result in excessive material thickness and hinder bracket visibility and positioning verification during clinical bonding.

### 2.3. Tray Fabrication and Clinical Bonding

The transfer trays used in this study were fabricated from a rigid resin material (Surgical Guide, Ludent, Anyang, Republic of Korea), specifically designed for dental applications. This material has a Shore D hardness of 67, indicating high rigidity, and an elongation at break of approximately 12.3%, suggesting limited flexibility before fracture. Additionally, the material is transparent, which facilitates effective light-curing of the adhesive during bracket bonding.

**Figure 2:**
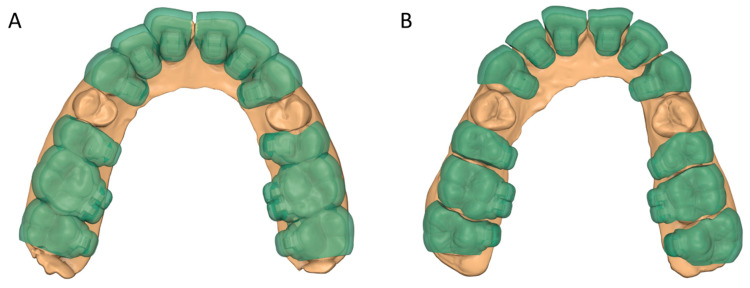
Three-dimensionally printed indirect bonding trays for lingual brackets: (**A**) grouped trays; (**B**) individual trays.

Patients were randomly assigned to one of two groups, a group receiving lingual brackets bonded with grouped transfer trays (GT) and a group receiving individual transfer trays (IT). For both groups, the tray thickness was 1.0 mm with a fully enclosed bracket lodgement design. In the GT group, three-tooth spans were used for both anterior and posterior teeth in premolar extraction cases, while two-tooth spans were used for posterior teeth in non-extraction cases due to there being four posterior teeth in these cases (Figure 2). To avoid uneven seating due to the potential bending of the rigid material during polymerization shrinkage, trays with four-tooth spans were not fabricated. Tray segmenting was performed using a 3D processing application (Medit Design version 2.1, Medit, Seoul, Republic of Korea).

Trays were 3D-printed using a digital light processing (DLP) printer (Photon D2, Anycubic, Shenzhen, China) with a build angle of 110° to ensure adequate support of the gingival wall of the bracket lodgements. Following printing, supports were removed, and the trays were cleaned with isopropyl alcohol. Post-curing was then performed with an ultraviolet lamp (Wash & Cure 3, Anycubic, Shenzhen, China) according to the manufacturer’s instructions. Any undercuts were removed using burs to facilitate tray removal after bonding. Lingual brackets (ADB, Medico, Seoul, Republic of Korea) were placed into the lodgements, and the trays with brackets were fitted on the study models to check for any remaining undercuts or misfits.

For clinical bonding, the lingual tooth surfaces were cleaned, etched with 37% phosphoric acid (FineEtch, Spident, Incheon, Republic of Korea) for 30 s, and primed (Assure Plus, Reliance, Itasca, IL, USA). An orthodontic adhesive was then applied to the bracket bases, followed by the placement of the tray onto the dental arches. The excess adhesive was removed, and the brackets were light-cured for 40 s (LedF, Woodpecker, Guilin, China). To facilitate tray removal, the tray material surrounding the brackets was reduced using burs and polishers (One Gloss, Shofu, Tokyo, Japan).

### 2.4. Data Acquisition

**Figure 3:**
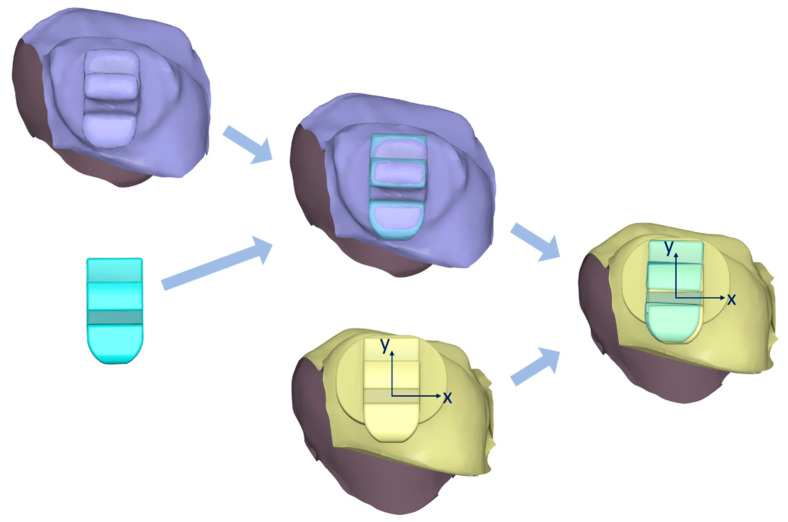
Measurement of bracket transfer errors. x, mesiodistal; y, occlusogingival.

Following bracket bonding, the dental arches were scanned with the same intraoral scanner. These scans served as the target data for comparison with the reference data, which consisted of the virtual study models with planned bracket positions. Reference and target data were imported into a 3D processing program (Medit Design version 2.1) to compare the actual bracket positions with the planned positions (Figure 3). For both the reference and target datasets, each tooth with its corresponding bracket was individually segmented, excluding gingival structures. A virtual bracket body was then aligned with the scanned bracket on the target data using a best-fit alignment method to facilitate accurate measurements. Subsequently, the target dataset was superimposed onto the reference dataset based on the dental crowns alone, without including the brackets in the superimposition process.

Linear measurements, including mesiodistal, buccolingual, and occlusogingival dimensions, were recorded as the translation of the bracket body’s center along the corresponding axes of the bracket slot. Angular measurements, including rotation, tip, and torque, were assessed as the angular deviation of the bracket body projected onto the planes formed by two corresponding axes. All deviations were quantified using 3D inspection software (Meshmixer version 3.5; Autodesk, San Francisco, CA, USA). Teeth with severe displacement that prevented bracket placement or resulted in loose brackets within the trays were excluded from the study. Additionally, brackets with bond failures during tray removal or those with excessive distortion from scan errors that precluded measurement were excluded from the analysis.

### 2.5. Statistical Analysis

To assess inter-observer reliability, a second observer independently re-measured a random sample of 24 brackets, and the intraclass correlation coefficient (ICC) was calculated. The normality of the variables was confirmed using the Shapiro–Wilk test. Brackets were categorized into three types including anterior, premolar, and molar. For each group and bracket type, transfer errors were reported as means and standard deviations for each dimension. One-sided *t*-tests were conducted to determine whether bracket errors were significantly less than 0.5 mm for linear measurements and 2° for angular measurements. These thresholds represent generally accepted limits for bracket transfer accuracy, as established in previous studies [2,6,11]. Two-sided *t*-tests were used to detect any significant differences in transfer errors between the two groups for each dimension and bracket type. All statistical tests were conducted with a significance level of 0.05.

## 3. Results

The two study groups, comprising 11 patients each, were comparable in terms of their demographic characteristics (Table 1). No statistically significant differences were observed between the GT and IT groups with respect to age, sex distribution, extraction versus non-extraction treatment plans, or angle classification (*p* > 0.05). Similarly, the groups exhibited comparable arch-length discrepancies (*p* = 0.828). While the bonding time for the IT group was slightly longer than that of the GT group, this difference was not statistically significant (*p* = 0.098). Two bonding failures occurred in the GT group, specifically on an upper lateral incisor and a lower second molar, and these teeth, along with those affected by scan errors, were excluded from the final analysis, leaving 274 teeth in the GT group and 272 in the IT group. Inter-observer reliability for the measurements was high, with ICCs of 0.923 for linear dimensions and 0.904 for angular dimensions.

In the GT group, mean transfer errors for the mesiodistal, buccolingual, occlusogingival, rotation, tip, and torque dimensions were 0.07 mm, 0.10 mm, 0.12 mm, 1.20°, 1.47°, and 2.24°, respectively (Table 2 and Table 3). With the exception of tip transfer errors for premolars, bracket transfer errors were significantly lower than the clinically accepted thresholds for all linear and angular measurements across all tooth types. Torque transfer errors, however, were not significantly lower than the accepted threshold for any tooth type.

In the IT group, the corresponding mean transfer errors were 0.10 mm, 0.12 mm, 0.13 mm, 1.47°, 1.73°, and 2.90°. Bracket transfer errors were significantly lower than the clinically accepted thresholds for mesiodistal, buccolingual, occlusogingival, and rotation for all tooth types, with the exception of premolar bracket rotation. For tip dimension, only molar bracket transfer errors were significantly lower than the threshold. Similarly to the GT group, torque transfer errors in the IT group were not significantly lower than the accepted threshold for any tooth type.

In general, angular transfer errors were greater than linear ones, with torque being the least accurately transferred dimension. No specific trends were observed regarding tooth type within each group. For the total sample, transfer errors were significantly lower than the accepted thresholds for all linear measurements and for rotation and tip in both groups. However, torque transfer errors were not significantly within the 2° threshold.

**Figure 4:**
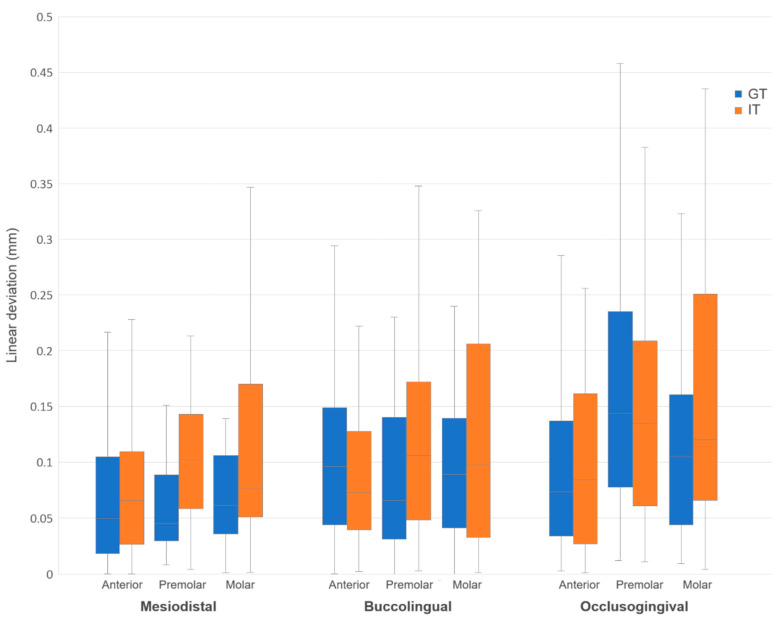
Box and whisker plot comparing linear transfer errors of grouped and individual trays.

**Figure 5:**
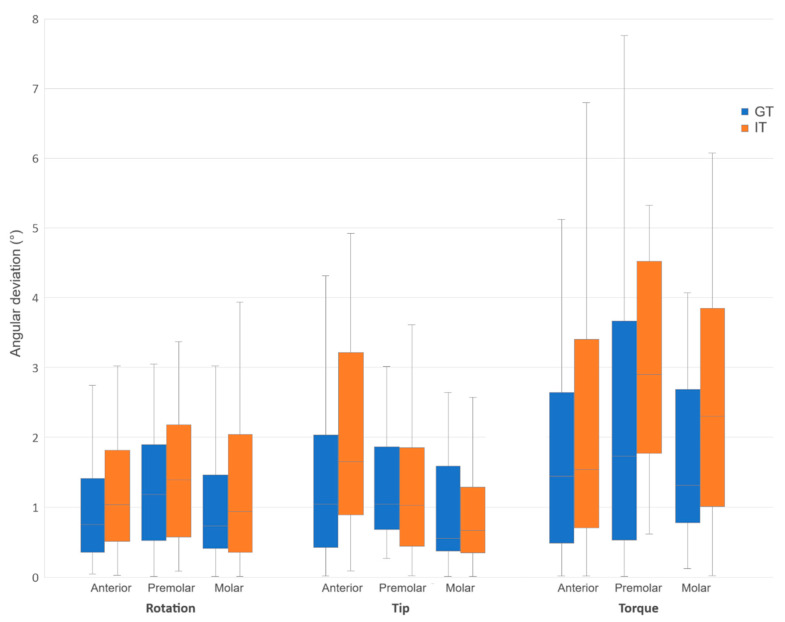
Box and whisker plot comparing angular transfer errors of grouped and individual trays.

Comparing the GT and IT groups directly, the GT group generally exhibited lower bracket transfer errors across most measured dimension and tooth type combinations (Figure 4 and Figure 5). However, statistically significant differences were observed only in the mesiodistal dimension for premolars (*p* = 0.004), in the tip dimension for anterior teeth (*p* = 0.007), and in the mesiodistal (*p* = 0.009) and torque (*p* = 0.044) dimensions for the total sample (Table 4).

## 4. Discussion

This study investigated the in vivo transfer accuracy of lingual brackets using AI-assisted, 3D-printed indirect bonding trays and compared the performance between grouped and individual tray designs. The findings partially rejected the null hypotheses: while most bracket transfer errors were within clinically acceptable thresholds, torque deviations exceeded the threshold in both tray types. Moreover, grouped trays demonstrated significantly better accuracy than individual trays in several dimensions, though not across all measured parameters or tooth groups.

In this study, artificial intelligence was applied during the tray design phase to automatically generate tray outlines and internal geometry based on tooth morphology, thereby reducing manual design time and enhancing standardization. Given the need for precise tray fabrication, a DLP printer was utilized in this study to ensure high printing accuracy [12]. A rigid resin material was used for tray printing to minimize tray distortion under finger pressure and achieve a more passive fit, especially for individual trays. Additionally, initial laboratory trials with flexible resin materials were unsuccessful due to excessive bracket looseness within the lodgements. In the study by Palone et al., which utilized 3D-printed flexible trays, wax and silicone material were necessary to secure the brackets within the trays, thereby increasing the complexity of the procedure [13]. Furthermore, the printing and post-processing of flexible resins are more difficult than those of rigid resins due to the higher viscosity of the former [1].

The bonding time in this study was significantly longer than that reported in previous studies. Anh et al., using vacuum-formed trays for lingual brackets, reported a bonding time of 42.3 min, while Czolgosz et al., using 3D-printed flexible trays for labial brackets, reported a bonding time of 25.7 min [6,14]. This discrepancy may be attributed to the additional time required for removing excess tray material around the brackets. Furthermore, the tray material tended to adhere to the orthodontic adhesive, necessitating additional cleaning effort. While the rigid resin material may contribute to extended chair time due to increased removal complexity, it also provides superior dimensional stability, ensuring precise bracket placement. This trade-off between accuracy and bonding efficiency should be considered in clinical applications. Further research could explore optimized tray designs to reduce cleanup time while maintaining high transfer precision. For example, modifying the tray geometry to minimize excess material surrounding the bracket could facilitate easier removal. Designing perforations or strategic cutouts around the bracket location may prevent adhesive adherence to the tray, streamlining the cleanup process.

The trend of higher transfer accuracy observed with grouped trays supports the hypothesis that a greater number of anatomical landmarks contribute to reduced transfer errors, even with the reduced inter-bracket distance inherent to lingual orthodontics. This enhanced accuracy is particularly evident in the mesiodistal and torque dimensions. The improved mesiodistal accuracy may be attributed to the potential for mesiodistal displacement with individual trays, especially for premolars, which have a rounded morphology. The enhanced torque accuracy may be due to the grouped tray’s resistance to buccolingual rotation, afforded by the curvature of the tooth segments. The significant differences in tip measurements of anterior teeth between the two tray types could be explained by the dependence of tray angulation on the position of finger pressure during seating. This pressure can induce mesial or distal tipping, particularly for canines, which have a conical shape, and for some lateral incisors with reduced sizes.

The generally higher angular transfer errors compared to linear errors were consistent with previous studies on both lingual and labial brackets [2,3,6]. This inherent characteristic may be attributed to the poor definition of bracket edges due to the rounding effect during scanning, which can lead to inaccuracies in determining the bracket axis during measurement. The lower transfer accuracy of torque, in particular, aligns with the findings from previous studies [2,3,6,10]. This is possibly due to the high dependence of torque accuracy on finger pressure during tray seating and the increased susceptibility of brackets to buccolingual rotation within their lodgements if the fit is not absolutely perfect.

The high variability observed in certain dimensions in this study, as reflected by the length of some error bars, warrants further discussion. Several factors may contribute to greater variability in torque transfer. Individual differences among patients, tooth anatomies, and crown inclination could play a role. Other contributing factors include tray fit, scanning limitations, and clinician technique during tray seating. A rigid tray material or uneven finger pressure may lead to bracket misalignment, contributing to the high variability observed in the measurement results. Recent advancements in noise reduction techniques, such as Transformer-based deep networks for medical image denoising, may offer insights into mitigating scanning-related inaccuracies in 3D dental imaging [15]. Furthermore, the latest research on the automated analysis of medical images highlights promising directions for developing AI-driven automated diagnostic and evaluative tools, which could similarly enhance tray design and bracket positioning accuracy in orthodontics [16].

The transfer accuracy of all linear dimensions and rotation achieved with grouped trays in this study was comparable to that reported in a previous study by Anh et al. on the transfer accuracy of lingual brackets using double vacuum-formed trays [6]. That study reported errors of 0.06 mm, 0.09 mm, 0.12 mm, and 1.28° for the mesiodistal, buccolingual, occlusogingival, and rotation dimensions, respectively. However, tip and torque transfer with grouped trays in the present study were slightly more accurate compared to the 1.73° and 2.96° reported in the previous study. These findings could be attributed to the greater number of steps involved in fabricating vacuum-formed trays, including model 3D printing and vacuum forming, which may lead to an accumulation of errors. Additionally, the use of a rigid resin in the present study may have contributed to higher dimensional stability and, consequently, greater accuracy.

Comparing the results of the present study with those of other studies on the transfer accuracy of 3D-printed grouped flexible trays for labial brackets reveals some variation. The in vivo study by Niu et al. reported comparable mesiodistal and rotation errors, of 0.07 mm and 1.22°, respectively, but greater buccolingual, occlusogingival, tip, and torque errors, of 0.13 mm, 0.19 mm, 2.25°, and 3.14°, respectively [2]. Bachour et al. reported similar buccolingual errors, 0.10 mm, but greater errors in the remaining dimensions, of 0.10 mm, 0.18 mm, 2.47°, 2.00°, and 2.55° for mesiodistal, occlusogingival, rotation, tip, and torque, respectively [11]. The flexibility of the indirect bonding trays in these previous studies might have contributed to their lower transfer accuracy.

However, an in vitro study by Hoffmann et al. reported lower angular transfer errors, of 0.41°, 0.50°, and 0.66° for rotation, tip, and torque, respectively [17]. This higher accuracy might be attributed to the standardized conditions under which they were conducted, eliminating the confounding effects of limited intraoral access and saliva. In contrast, the in vivo study by Palone et al. reported even better accuracy across all dimensions, of 0.02 mm, 0.01 mm, 0.01 mm, 0.46°, 0.38°, and 0.36° for mesiodistal, buccolingual, occlusogingival, rotation, tip, and torque, respectively [13]. These findings may stem from more stable bracket positions within the trays due to the utilization of wax and silicone materials to secure the brackets, as well as potential differences in measurement approaches. Regarding 3D-printed grouped rigid trays, Schwarzler et al. reported lower mesiodistal and angular errors of 0.03 mm and 0.26°, similar occlusogingival errors of 0.12 mm, and greater buccolingual errors of 0.16 mm [9].

In the context of individual trays, a previous in vivo study by Schubert et al. measured the transfer accuracy of lingual brackets using individual trays fabricated with the traditional analog method [5]. That study reported comparable linear transfer errors of 0.12 mm, 0.13 mm, and 0.10 mm in the mesiodistal, buccolingual, and occlusogingival dimensions, respectively. However, rotation and tip errors, of 2.29° and 3.21°, respectively, were greater in the previous study, potentially due to the smaller occlusal pad of the trays used. In contrast, torque errors, 2.20°, were slightly lower, possibly because of a better bracket fit within prefabricated lodgements. Another study, an in vitro investigation by Kim et al., measured labial bracket transfer accuracy with individual trays [18]. They reported slightly lower mesiodistal, buccolingual, and rotation errors of 0.07 mm, 0.10 mm, and 1.28°, respectively, comparable tip and torque errors of 1.53° and 3.01°, respectively, and greater occlusogingival errors of 0.17 mm.

The lower transfer accuracy of individual trays observed in this study may explain the possible need for wire bending during the finishing stage in lingual orthodontic patients indirectly bonded with analog individual trays, as this compensates for potential inaccuracies in bracket placement. In a case study by Albertini et al., transfer error was evident in the differing adhesive thicknesses between upper central incisor brackets, leading to a torque error [19]. This manifested as a retroclined, lingually displaced, and extruded upper left central incisor despite its pretreatment normal alignment. This iatrogenic malalignment was only corrected after inserting an auxiliary torque spring.

This study has several limitations. First, the application of AI was limited to the tray design phase, with no involvement in the orthodontic setup, measurement, or evaluation of bracket transfer accuracy due to the lack of such functions in the available software. Future research may expand the role of AI beyond design into diagnostic and evaluative domains; for example, by enabling automatic deviation measurements directly from intraoral scan data or by developing predictive models to assess bracket positioning risk based on morphological or arch-related characteristics. Second, the accuracy of 3D-printed individual transfer trays may be inferior to that of analog individual trays fabricated on plaster dies. This potential inferiority is attributed to the superior fit of analog trays, as anatomical details may be lost during the digital scanning and printing process. However, the accuracy of analog trays was not evaluated in this study. Third, while this study aimed to compare the transfer accuracy between grouped and individual trays, the bonding procedure for each tray type was performed on separate patients, which may introduce variability due to differences in arch conditions, crowding patterns, tooth anatomy, and host responses. Ideally, a repeated-bonding or split-mouth design could have minimized these confounding factors, but ethical and practical constraints limited this approach. However, to mitigate this limitation, patient assignment was randomized, and statistical analysis confirmed that both groups were comparable in terms of demographic characteristics, treatment plans, and arch-length discrepancies. Future studies employing a within-subject or split-mouth design may further enhance the accuracy of the comparisons between tray types. Furthermore, although the sample size calculation was appropriately based on torque measurements, its adequacy for detecting differences in other dimensions remains uncertain. With only 11 patients per group, statistical power may have been insufficient for secondary outcomes, potentially explaining why certain differences showed trends without reaching significance. Future studies with larger sample sizes could improve reliability and ensure more robust comparisons across multiple parameters.

### Clinical Relevance

The findings of this study provide practical guidance for orthodontists when choosing and applying indirect bonding trays, especially for lingual brackets. Both grouped trays and individual-tooth trays were shown to achieve clinically acceptable accuracy in transferring brackets to the teeth, with five out of the six measured dimensions falling within clinically accepted thresholds. This means that in everyday practice, either type of tray can be used reliably to place brackets in the planned positions. However, our results indicate a slight advantage with grouped trays in terms of placement precision for certain directions. In simpler terms, using one larger tray that fits over multiple teeth at once can make bracket placement a bit more precise, because the tray rests on several teeth and is less likely to shift during bonding.

In practice, a grouped tray design may be preferable for the initial bonding of an entire arch or segment of teeth, as long as the patient’s dental anatomy allows the tray to fully seat. On the other hand, individual trays are still very useful. For cases with significant crowding or unique tooth shapes, where a full-section tray might not fit perfectly, delivering one bracket at a time with small, individual trays can be easier and still accurate. Individual trays are also convenient for bonding a single bracket (for example, when reattaching a bracket mid-treatment or placing a bracket after space creation for a severely displaced tooth) without having to remake a whole arch tray. In using either type of tray, clinicians should pay attention to how they seat the tray: applying even, firm pressure across the tray is important to avoid any tilt or misalignment. Finally, incorporating the AI-assisted design and 3D printing workflow as described in our study can streamline the tray fabrication process. This digital approach can save laboratory time and produce trays that fit precisely, ultimately helping clinicians bond brackets more efficiently while maintaining a high level of accuracy in bracket placement.

## 5. Conclusions

Both grouped and individual designs of AI-assisted, 3D-printed rigid indirect bonding trays demonstrated the clinically acceptable transfer accuracy of lingual brackets across all linear dimensions and most angular dimensions. Grouped trays generally exhibited higher precision, particularly in mesiodistal and torque positioning. However, the accuracy of torque transfer remains a challenge for both tray types. Although AI was not used in the measurement or analysis of bracket accuracy, its application in tray design contributed to a streamlined and consistent digital workflow. These findings support the integration of AI-assisted design tools in orthodontic practice to enhance the efficiency of appliance fabrication while maintaining clinical accuracy. Additionally, grouped trays are recommended for initial bracket placement, while individual trays offer a reliable option for subsequent bonding procedures, such as replacing failed brackets or bonding teeth that were not initially bonded.

## Figures and Tables

**Table 1:** Demographic characteristics of the study groups.

Characteristic	Grouped Trays (*n* = 11)	Individual Trays (*n* = 11)
Age	29.15 ± 7.35	27.58 ± 5.22
Sex		
Male	5 (45.5%)	4 (36.4%)
Female	6 (54.5%)	7 (63.6%)
Extraction		
Non-extraction	5 (45.5%)	4 (36.4%)
Extraction	6 (54.5%)	7 (63.6%)
Angle Classification		
Class I	5 (45.4%)	6 (54.5%)
Class II	4 (36.4%)	3 (27.3%)
Class III	2 (18.2%)	2 (18.2%)
Arch-length discrepancy (mm)	3.15 ± 3.45	3.44 ± 2.48
Bonding Time (min)	83.29 ± 12.41	95.15 ± 17.37
Teeth		
Included in study	278	276
Excluded (bond failure)	2	0
Excluded (scan error)	2	4
Included in analysis	274	272

**Table 2:** Bracket transfer errors of grouped trays for each tooth type and the total sample. *, bracket errors were significantly less than 0.5 mm and 2°, as indicated by one-sided *t*-tests.

Grouped Trays	Anterior (*n* = 62)	Premolar (*n* = 32)	Molar (*n* = 43)	Total (*n* = 137)
Mesiodistal (mm)	0.07 ± 0.06 *	0.06 ± 0.05 *	0.08 ± 0.07 *	0.07 ± 0.06 *
Buccolingual (mm)	0.11 ± 0.08 *	0.09 ± 0.09 *	0.09 ± 0.07 *	0.10 ± 0.08 *
Occlusogingival (mm)	0.10 ± 0.08 *	0.17 ± 0.11 *	0.12 ± 0.11 *	0.12 ± 0.10 *
Rotation (°)	1.12 ± 1.14 *	1.53 ± 1.45 *	1.06 ± 0.90 *	1.20 ± 1.16 *
Tip (°)	1.47 ± 1.37 *	1.78 ± 1.93	1.25 ± 1.49 *	1.47 ± 1.56 *
Torque (°)	2.23 ± 2.63	2.80 ± 2.95	1.83 ± 2.22	2.24 ± 2.60

**Table 3:** Bracket transfer errors of individual trays for each tooth type and the total sample. *, bracket errors were significantly less than 0.5 mm and 2°, as indicated by one-sided *t*-tests.

Individual Trays	Anterior (*n* = 62)	Premolar (*n* = 31)	Molar (*n* = 43)	Total (*n* = 136)
Mesiodistal (mm)	0.08 ± 0.06 *	0.11 ± 0.08 *	0.14 ± 0.22*	0.10 ± 0.14 *
Buccolingual (mm)	0.10 ± 0.10 *	0.15 ± 0.14 *	0.12 ± 0.10*	0.12 ± 0.11 *
Occlusogingival (mm)	0.11 ± 0.10 *	0.15 ± 0.11 *	0.16 ± 0.11 *	0.13 ± 0.11 *
Rotation (°)	1.34 ± 1.29 *	1.92 ± 2.26	1.34 ± 1.42 *	1.47 ± 1.60 *
Tip (°)	2.33 ± 2.06	1.60 ± 1.66	0.96 ± 0.93 *	1.73 ± 1.78 *
Torque (°)	2.65 ± 3.00	3.91 ± 3.31	2.54 ± 1.94	2.90 ± 2.82

**Table 4:** Results of two-sided *t*-tests comparing bracket transfer errors between the two groups. *, *p* < 0.05.

	Anterior	Premolar	Molar	Total
Mesiodistal	0.552	0.004 *	0.091	0.009 *
Buccolingual	0.666	0.058	0.161238	0.120
Occlusogingival	0.406	0.567	0.147231	0.291
Rotation	0.309	0.424	0.266734	0.102
Tip	0.007 *	0.702	0.289779	0.201
Torque	0.411	0.167	0.115851	0.044 *

## Data Availability

The data presented in this study are available on request from the corresponding author due to privacy restrictions.

## Abbreviations

The following abbreviations are used in this manuscript:

3D	Three-dimensional
AI	Artificial intelligence
DLP	Digital light processing
GT	Grouped tray
ICC	Intraclass correlation coefficient
IT	Individual tray
STROBE	Strengthening the Reporting of Observational Studies in Epidemiology
